# Predicting the effect of chemicals on fruit using graph neural networks

**DOI:** 10.1038/s41598-024-58991-y

**Published:** 2024-04-08

**Authors:** Junming Han, Tong Li, Yun He, Ziyi Yang

**Affiliations:** 1 College of Food Science and Technology, Yunnan Agricultural University, Kunming, 650201 China; 2 Yunnan Agricultural University, Kunming, 650201 China; 3 College of Big Data, Yunnan Agricultural University, Kunming, 650201 China; 4 College of Agronomy and Biotechnology, Yunnan Agricultural University, Kunming, 650201 China

**Keywords:** Neural networks, Computational chemistry, Artificial intelligence, Food quality, Chemical engineering, Mechanical engineering

## Abstract

The neural network method is a type of machine learning that has made significant advances over the past few years in a variety of fields, particularly text, speech, images, videos, etc. In areas where data is unstructured, traditional machine learning has not been able to surpass the ’glass ceiling’; therefore, researchers have turned to neural networks as auxiliary tools to achieve significant breakthroughs or develop new research methods. An array of computational chemistry challenges can be addressed using neural networks, including virtual screening, quantitative structure-activity relationships, protein structure prediction, materials design, quantum chemistry, and property prediction, among others. This paper proposes a strategy for predicting the chemical properties of fruits by using graph neural networks, and it aims to provide some guidance to researchers and streamline the identification process.

## Introduction

Among the most important areas of chemical research is the study of properties of chemical substances, due to its importance in understanding their essence and characteristics, promoting the advancement of chemical science, and solving practical problems. The chemical substances in foods are particularly relevant to the health of people, particularly in the food industry.

To determine whether a chemical will affect the quality of food, acute and chronic toxicity tests, genotoxicity studies, metabolic studies, and other complex studies are usually required. The continuous development of computer technology has prompted many researchers to use computational methods to predict chemical properties. These methods include linear regression, decision trees, support vector machines, random forests, and other machine learning algorithms. With the continuous development of computer technology, many researchers are constantly trying to use computational methods to solve the problem of predicting chemical properties, like linear regression, decision tree, support vector machine, random forest and other machine learning algorithms, but these algorithms require domain experts to participate and provide a lot of professional knowledge, and if the nonlinear transformations are chosen poorly, its complexity will increase exponentially^1^.

Recently, artificial intelligence and machine learning have demonstrated their potential for predicting chemistry and synthesizing small molecules^2^. along with algorithm upgrades and GPU-accelerated computing. The development of artificial intelligence has allowed at least some operations that require strong disciplinary background to be replaced by computers. Consequently, many teams use computers in the study of biology and chemistry to solve a wide variety of problems.

Jumper et al.^3^ from the DeepMind team pointed out that proteins are crucial to life, and understanding their structure can promote a systematic understanding of their functions. Therefore, they adopted deep learning algorithms and studied the emergence of AlphaFold2, which improved the accuracy of protein structure prediction to over 90%, with only one atomic width difference from the actual protein structure, truly solving the problem of protein folding.

Jha et al.^4^ proposed a deep neural network model called ElemNet, which uses artificial intelligence to automatically capture physical and chemical interactions and similarities between different elements, making it possible to predict the properties of materials with better accuracy and speed. The speed and best-in-class accuracy of ElemNet allows performing fast and stable screening for new material candidates in a huge combinatorial space and predicts that thousands of chemical systems may contain as yet undiscovered compounds.

Goh et al.^1^ provided an introductory overview of the theory of deep neural networks and their unique properties compared to traditional machine learning algorithms used in chemical informatics. By summarizing the various emerging applications of deep neural networks, its universality and wide applicability are emphasized to address a wide range of challenges in this field, including quantitative structure-activity relationships, virtual screening, protein structure prediction, quantum chemistry, material design, and attribute prediction. It is expected that deep learning algorithms will become valuable tools in computational chemistry.

AlQuraishi et al.^5^ proposed mathematical equations for specific fields of natural sciences and general machine learning models trained on experimental data, which have an increasing impact on molecular and cellular biology. They also demonstrated relevant biological experiments, demonstrating that biophysics and functional genomics have made significant progress with the help of machine learning.

Sun et al.^6^considered extracting informative representations of molecules using graph neural networks is crucial in AI-driven drug discovery, and they have been successful in terms of training objectives, data segmentation methods, input features, pre-training dataset sizes and GNN architectures. Duvenaud et al.^7^present architecture that generalizes standard molecular feature extraction methods based on circular fingerprints. Gilmer et al.^8^provider a single common framework we call Message Passing Neural Networks (MPNNs), and using MPNNs we demonstrate state of the art results on an important molecular property prediction benchmark. Graph neural networks have recently had great success in predicting the quantum mechanical properties of molecules, Gasteiger et al.^9^ propose directional message passing, in which we embed the messages passed between atoms instead of the atoms themselves. Liu et al.^10^ propose spherical message passing (SMP) as a novel and powerful scheme for 3D molecular learning. SMP significantly reduces the complexity of training, allowing it to perform efficiently on large-scale molecules. Moreover, SMP is able to distinguish almost all molecular structures.

The rapid development of high-throughput technologies has made it possible to obtain a large amount of genomic data at a much lower cost, which requires the use of deep learning techniques for analysis. Thus Wang et al.^11^ identified two prominent issues at the intersection of genomics and deep learning at present: how to model the information flow from genomic DNA sequences to molecular phenotypes; How to use deep learning models to identify functional variations in natural populations and propose the core role of deep learning in future plant genomics research and crop genetic improvement.

With the latest advances in computational biology, high throughput next generation sequencing technology has become the defacto standard technique for gene expression research, including DNA, RNA, and proteins. As a promising technology with a significant impact on medical science and genome research, Khan et al.^12^ proposed a computing model based on parallel deep neural networks, which utilizes the advantages of parallel and distributed computing platforms to timely classify a large number of RNA sequences into piRNAs and non piRNAs. The performance of the proposed model was evaluated using dual performance indicators, compared to sequential methods, the proposed model improves computational speed by an order of magnitude without affecting the accuracy level.

Deciphering gene regulatory networks is a central problem in computational biology, and Liu et al.^13^ explored the use of a multimodal neural network to learn a predictive model of gene expression that includes both cis and trans-regulatory components. By modelling the stress response in the budding yeast Saccharomyces cerevisiae, the model achieves high performance and greatly outperforms other state-of-the-art approaches.

Predicting the spatial structure or function of biomolecules based on their sequences remains an important challenge in bioinformatics. When modelling biological sequences using traditional sequencing models, distance interactions, complex and variable outputs of tag structures and variable lengths of biological sequences usually have different solutions depending on the specific situation, thus Wu et al.^14^ proposed a unified deep learning architecture based on long-short-term memory or gated loop units to capture the interactions. The architecture designs optional reshape operators to accommodate the diversity of output labels and implements a training algorithm to support the training of sequence models capable of handling variable length sequences. The model is also validated on the prediction of protein residue interactions, one of the most difficult biological sequence modelling problems. The results show that the accuracy of residue interactions obtained for the model is 10% higher than commonly used methods in several widely used benchmark tests.

Fruit is a kind of food and it is an important part of the human diet, there contains essential minerals, vitamins, and dietary fiber^15^, so this paper proposes an efficient way to predicting the properties of chemicals in fruits and whether they affects the quality of fruits.

## Graph neural networks and molecule

**Figure 1:**
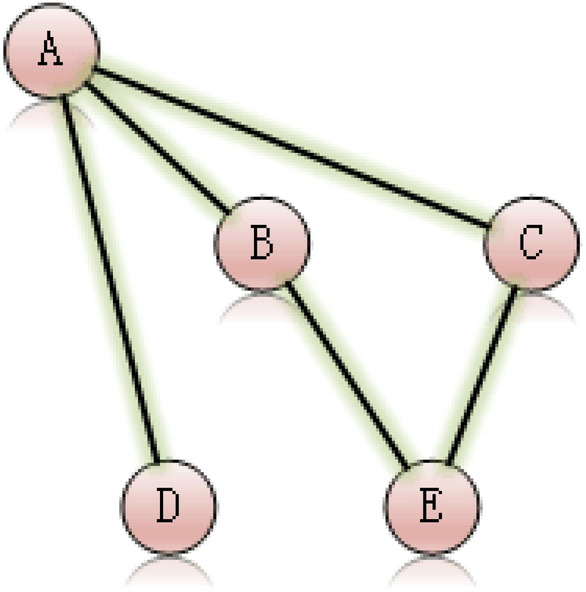
Example of a graph.

Graph is a typical structure in computer science. A graph represents the relations (edges) between a collection of entities (nodes), and is represented as G $=$ (*V*, *E*), that *V* is the set of nodes and *E* is the set of edges. As Fig. 1 shows, A, B, C, D and E are nodes, the relations between AB, AC, AD, BE and CE are edges.

**Figure 2:**
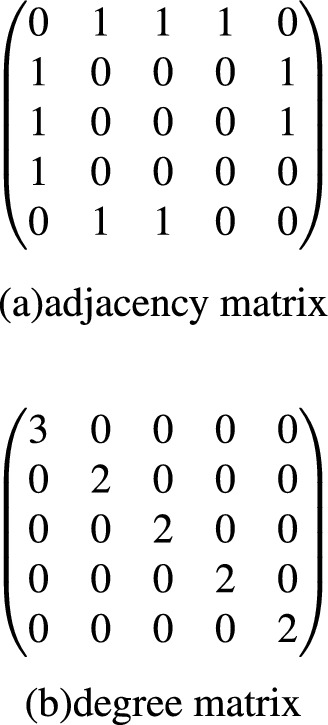
The adjacency matrix and degree matrix of Fig. 1.

A way of visualizing the connectivity of a graph is through its adjacency matrix and degree matrix. For a simple graph with node set *V* = v_1_, ..., v_n_, the adjacency matrix is a square n$\times$n matrix A such that its element A_ij_ is one when there is an edge from node u_i_ to node u_j_, and zero when there is no edge, and the degree matrix is a matrix which contains information about the degree of each node, that is, the number of edges attached to each vertex. Figure 2 shows adjacency matrix and degree matrix of Fig. 1.

**Figure 3:**
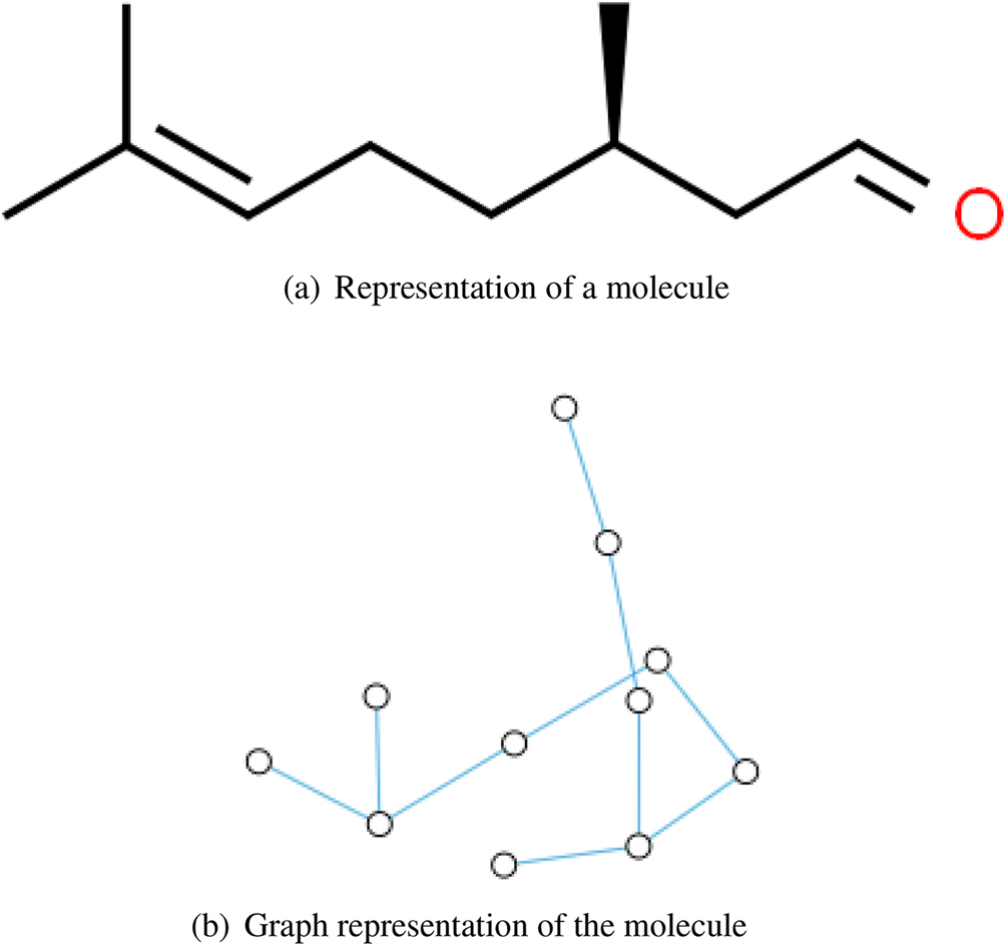
Examples of graph representation of a molecule.

In a molecule, atoms are represented as notes, and chemical bonds are represented as edges between the *i*th and *j*th atoms^16^, is as Fig. 3 shows.

**Figure 4:**
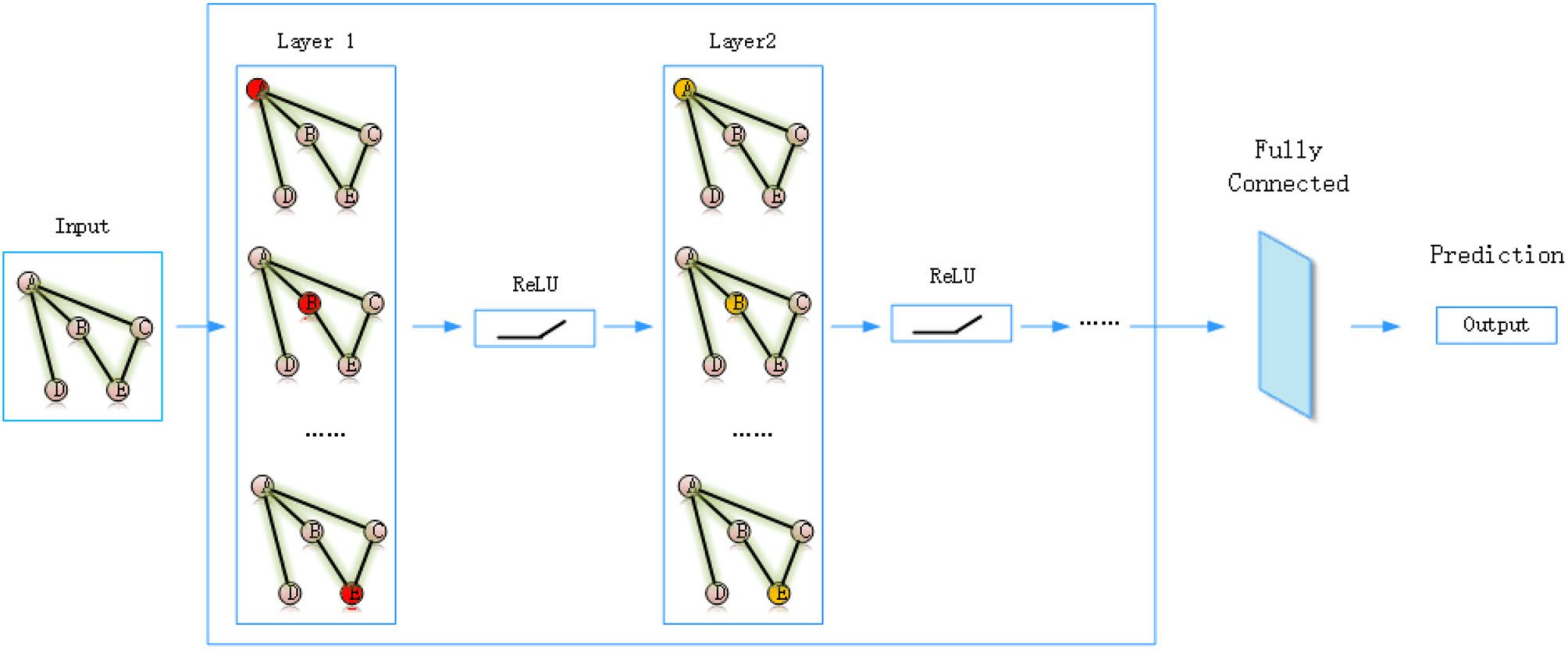
Architecture of graph neural networks.

The architecture of graph neural networks as Fig. 4 shows, the algorithm aggregate information about surrounding nodes and then updates the node information.

The feature vectors of each node in the graph are initialized at the beginning of the calculation. Fully Connected Layer is one of the many components of a multilayer perceptron applied to a neural network. In the field of deep learning, the network structure of a neural network model for classification tasks often ends with a fully connected layer, which is used to map the feature expression vectors obtained from the several feature extraction layers prior to this layer to the next layer. Multilayer perceptron is a common artificial neural network model, it consists of an input layer, several hidden layers, and an output layer, each layer containing several neurons, neurons are connected by weights, and each neuron calculates the weighted sum of its input and weight, then a non-linear transformation is performed through an activation function to output to the next layer of neurons. Finally, the output is predict the classification of graph.

**Figure 5:**
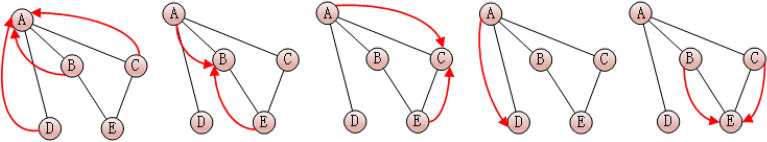
Example of updating adjacent node features.

As Fig. 5 shows, each node in the graph is updated with the features of neighbouring nodes during propagation.

**Figure 6:**
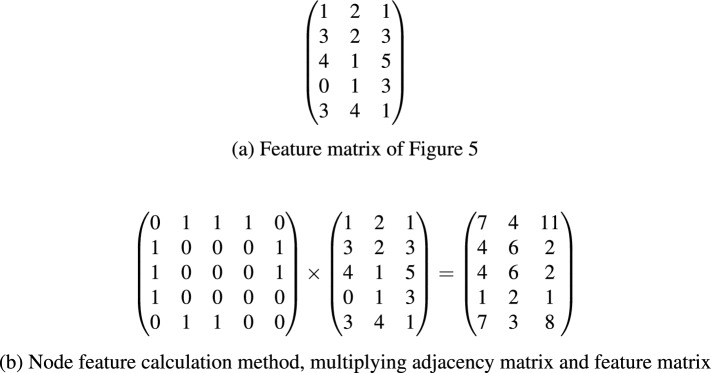
Feature matrix of and the node feature calculation method.

Assume that the feature matrix as Fig. 6 shows, (a) shows each row represents the feature vector of a node, and (b) shows the method of which each node gets the neighbouring.

The feature propagation formula between adjacent layers in graph neural networks as following formula show.

$$\begin{aligned} H^{(l+1)} = \sigma (\tilde{D}^{-\frac{1}{2}}\tilde{A}\tilde{D}^{-\frac{1}{2}}H^{(l)}W^{(l)}) \end{aligned}$$

$\tilde{A} = A\text {+}I$, A is the adjacency matrix of graph, and I is the identity matrix. $\tilde{D}$ is degree matrix of $\tilde{A}$. $W^{(l)}$ is the weight of *l*th layer. $H^{(l)}$ is the feature of *l*th layer.

## Dataset, evaluation metrics and parameters

**Figure 7:**
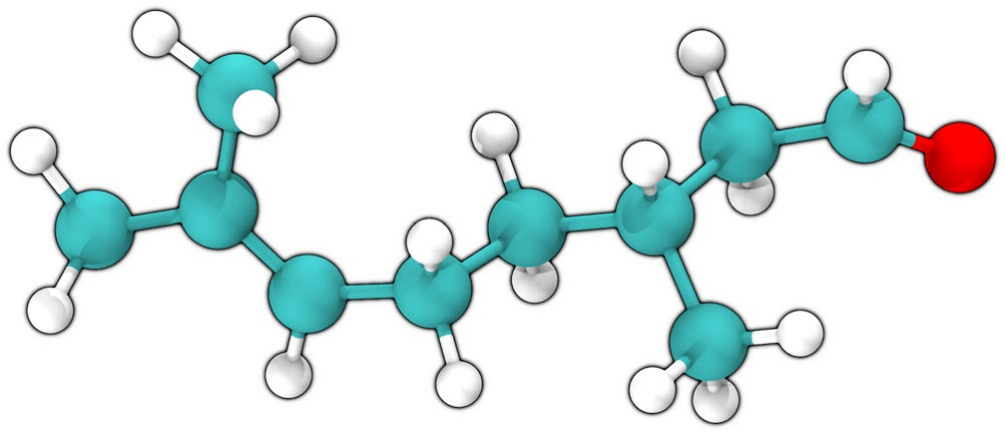
Three-dimensional representation of a molecule.

For a better performance of graph neural networks, the dataset used in this paper was collected from PubChem, which is an open source repository that includes chemical structures. Usually, a molecule can be described as a two-dimensional or three-dimensional form, however two-dimensional or three-dimensional are images, hardly represented by graph, so we used Simplified Molecular Input Line Entry System(SMILES) as input. For example, three dimension representation of the molecular formula $C_{10}H_{18}O$ as Fig. 7 shows, and the SMILES of $C_{10}H_{18}O$ is *C[C@H](CCC=C(C)C)CC=O*.

The dataset contains 174 small molecule compounds, these small molecule com-pounds have a positive effect on fruit quality, like Chlorophyll B, Xanthophyll, cyanidin chloride and so on. At the same time, we collected 174 small molecule compounds, which have no effect or negative effect on fruit quality. So the whole dataset contains 348 small molecule compounds.

In the machine learning field and specifically problem of statistical classification, a confusion matrix as Table 1 shows, is a specific table layout that allows visualization of the performance of an algorithm^17^, typically a supervised learning one (in unsupervised learning it is usually called a matching matrix). Each row of the matrix represents the in-stances in a predicted class, while each column represents the instances in an actual class^18^.

**Table 1:** Confusion matrix.

Predicted class	Actual class	
	Positive	Negative
Positive	True Positive	False Positive
Negative	False Negative	True Negative

According to the confusion matrix, almost all researchers use Precision, Recall, F1-Score, Receiver Operating Characteristic Curve (ROC) and Area Under Curve (AUC) to evaluate the models.

$$\begin{aligned} Pecision= & {} \frac{TP}{TP + FP} \\ Recall= & {} \frac{TP}{TP + FN} \\ False\ Positive\ Rate(FPR)= & {} \frac{FP}{FP + TN} \\ True\ Positive\ Rate(TPR)= & {} \frac{TP}{TP + FN} \\ F1= & {} \frac{2 \times Precision \times Recall}{Precision + Recall} \end{aligned}$$

TP represents true positive, i.e., the number of positive samples which are classified correctly. FP represents false positive, i.e., the number of negative samples which are classified incorrectly as positive ones. TN represents true negative, i.e., the number of negative samples which are classified correctly. FN represents false negative, i.e., the number of positive samples which are classified incorrectly as negative ones^19^. ROC is a curve, the values for the Y-axis and X-axis are True Positive Rate and False Positive Rate. AUC is the area enclosed by ROC and the X-axis.

The Table 2 shows parameters and its values used in this paper’s methods.

**Table 2:** The values of precision, recall, F1 and AUC.

Name	Value
Radius	1
Layer of graph	6
Layer of fully connected	10
Batch size	4
Learning rate	0.0001
Learning rate decay	0.99
Decay interval	10
Iteration	1000

## Results and experiments

This paper used cross-validation^20^, it is any of various similar model validation techniques for assessing how the results of a statistical analysis will generalize to an independent data set. Cross-validation includes resampling and sample splitting methods that use different portions of the data to test and train a model on different iterations. It is often used in settings where the goal is prediction, and one wants to estimate how accurately a predictive model will perform in practice. It can also be used to assess the quality of a fitted model and the stability of its parameters. Hold-Out is one method of cross-validation, Hold-Out is the division of the data set into two mutually exclusive sets, one for training, and another one for testing. So the dataset was divided into two parts randomly, 70% for training and, other 30% for testing.

The precision is the result of a model’s prediction, which is calculated over the entire positive samples predicted by the model, and it is defined as the probability of a truly positive sample among all positive samples predicted by the model; in other words, the precision rate describes the probability of a correct prediction among the samples predicted to be of a positive class.

**Figure 8:**
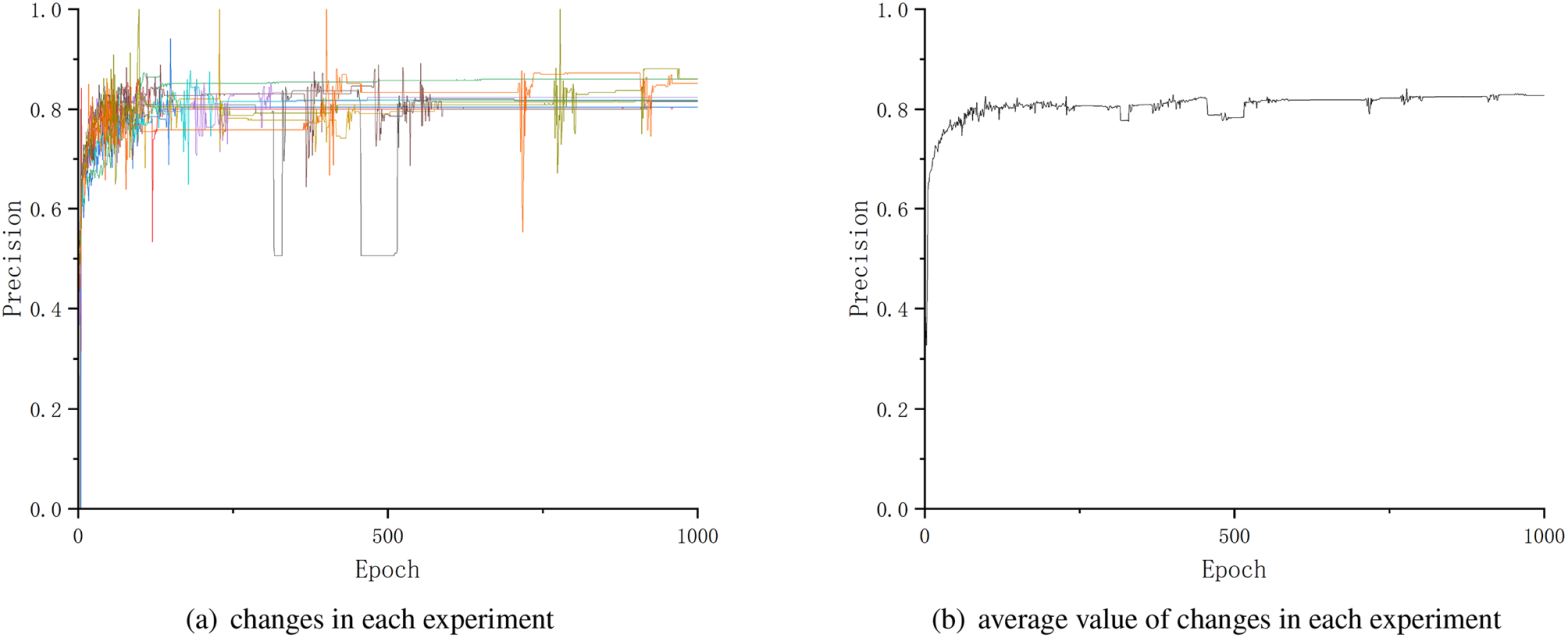
Changes of precision.

The Fig. 8 shows the change of precision in each experiment.

The recall expresses the probability of being correctly predicted by the model in all positive samples.

**Figure 9:**
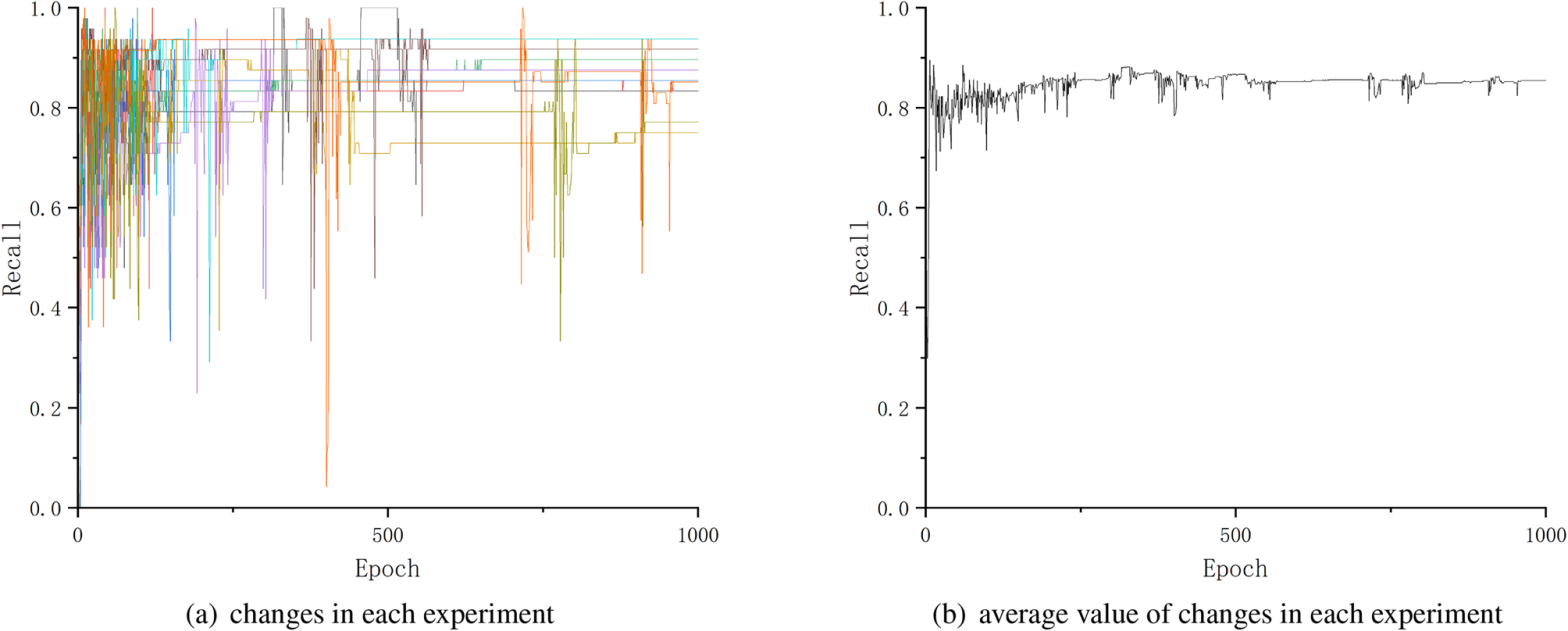
Changes of recall.

The Fig. 9 shows the change of recall in each experiment.

As can be seen from the definitions of precision and recall, these two metrics are con-tradictory. Usually, if the recall rate is to be increased, then the precision rate will decrease. For example, to increase the recall rate, we have to try to test as many samples as possible, but as the number of tested samples increases, the False Positive grows faster than the True Positive, so it leads to a decrease in the precision rate, and F-measure.

Therefore, F-Measure is used to balance precision and recall, also called weighted harmonic mean.

$$\begin{aligned} F-measure = \frac{(\alpha ^{2} + 1) \times precision \times recall}{\alpha ^{2} \times precision + recall} \end{aligned}$$

**Figure 10:**
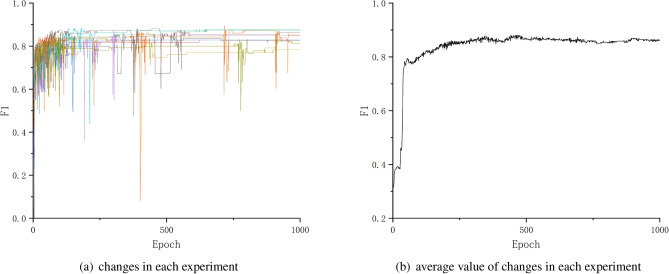
Change of F1.

If the experiment more concerned about recall, the larger value of a should be selected. Normally, the a is assigned a value of 1, which is F1. The Fig. 10 shows the change of F1 in each experiment.

As the Figs. 8, 9 and 10 shows, after about 500th testing, the precision, recall and F1 achieved higher values, and almost kept or achieved a little bit improvement in the subsequent testing, even if there are a few testing epochs where the values decreased.

**Figure 11:**
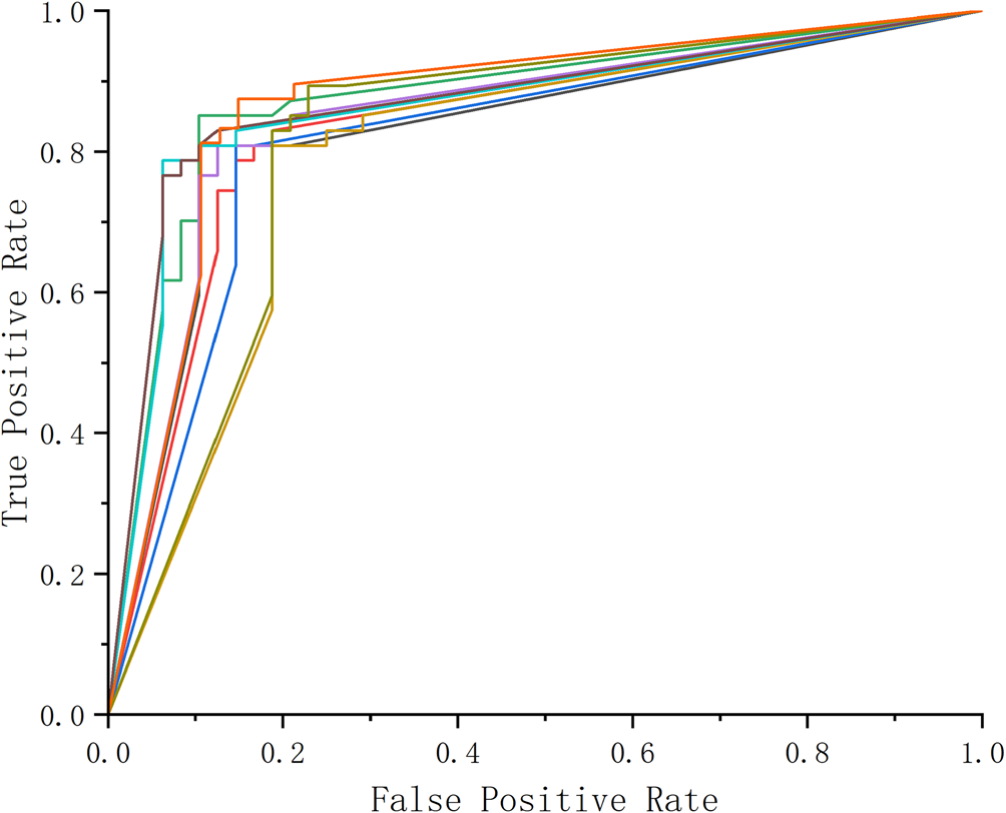
The receiver operating characteristic curve in each experiments.

A receiver operating characteristic curve, or ROC curve, is a graphical plot that illustrates the performance of a binary classifier model (can be used for multi class classification as well) at varying threshold values. The Fig. 11 shows the receiver operating characteristic curve in each experiments. The ROC curve is the plot of the true positive rate (TPR) against the false positive rate (FPR) at each threshold setting.

The Table 3 shows in each experiment, the values of precision, recall, F1 and AUC.

**Table 3:** The values of precision, recall, F1 and AUC.

No.	Precision (% )	Recall (% )	F1 %	AUC
1	81.63	83.33	82.47	0.8302
2	80.39	85.42	82.83	0.8333
3	80.39	85.42	82.83	0.8169
4	86.00	89.58	87.76	0.8754
5	82.35	87.50	84.85	0.8486
6	81.82	75.00	78.26	0.7945
7	81.82	93.75	87.38	0.8652
8	81.48	91.67	86.28	0.8712
9	86.05	77.08	81.32	0.8185
10	85.11	85.11	85.10	0.8703

**Figure 12:**
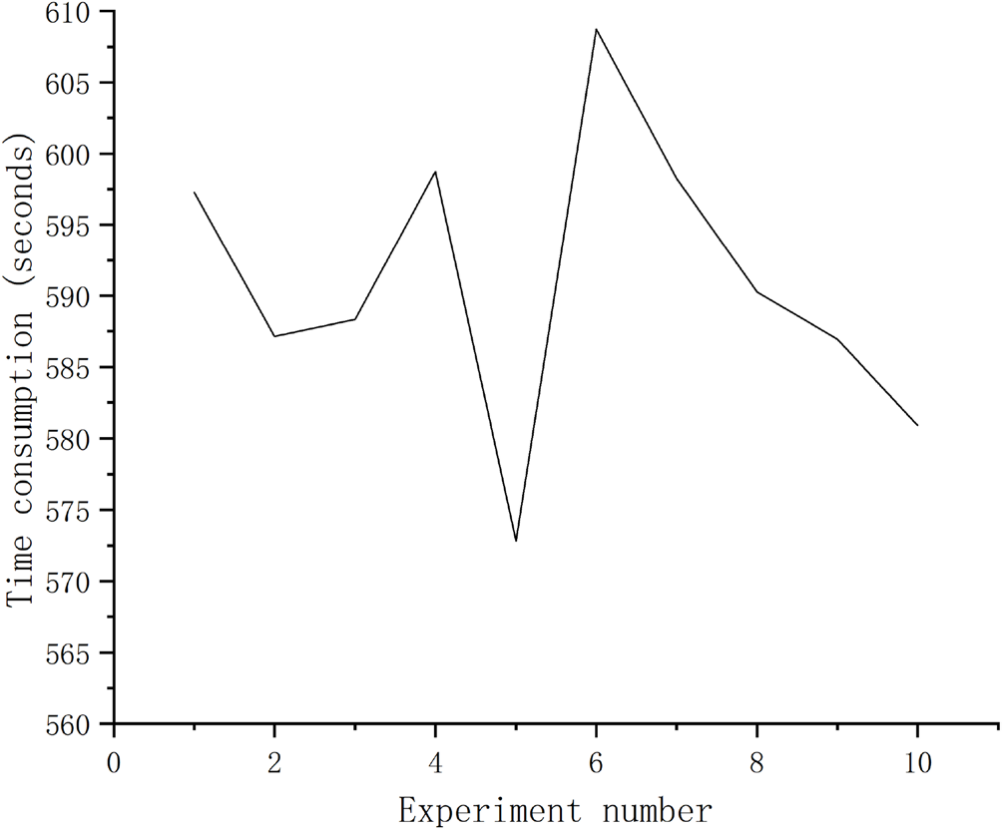
Time consumed for each experiment.

The Fig. 12 show time consumed for each experiment. The time consumed for ten experiments, maximum is 608.73 s, minimum is 572.82 s, and average is 590.94 s.

**Figure 13:**
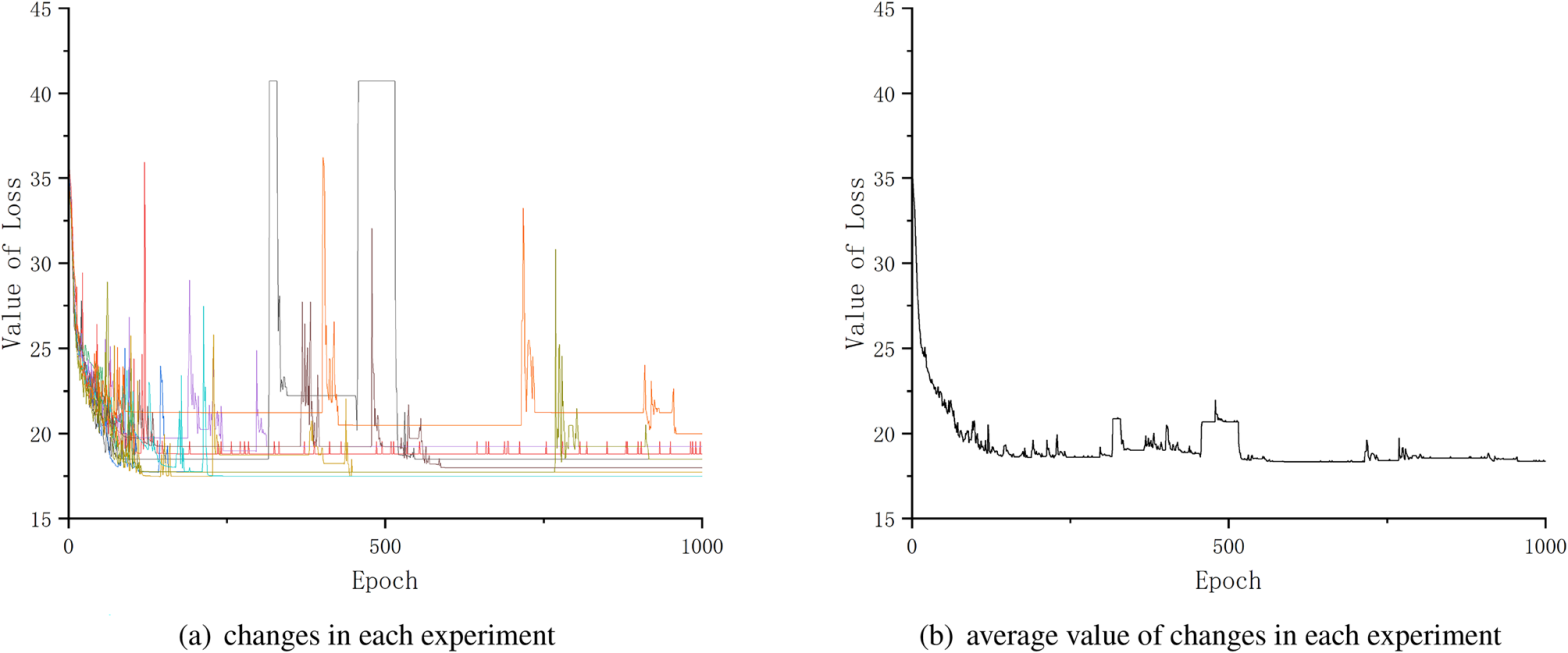
Change of loss value.

The loss function is mainly used in the training phase of the model, after each epoch of training data is fed into the model, the predicted value is output through forward propagation, and then the loss function calculates the difference between the predicted value and the true value, which is the loss value. After calculating the loss value, the model updates each parameter through backpropagation to reduce the loss between the true value and the predicted value (Fig. 13), so that the predicted value generated by the model is closer to the true value, to achieve the purpose of learning.

This article uses the mean squared error (MSE) as the loss function, which is defined as following formula show.

$$\begin{aligned} \text {MSE= }\frac{1}{m}\sum _{i=1}^m(y_i-\overset{\wedge }{{\text {*}}{y_i}})^2 \end{aligned}$$

m is the number of samples. $y_i$ is the real value. $\overset{\wedge }{{\text {*}}{y_i}}$ is the real value.

## Conclusions and future work

In this paper, graph neural networks are used to predict the properties of chemicals found in fruits. In this study, we selected a dataset and conducted experiments within it. According to the results of the experiments, the model we used exhibited good performance, with average precision, recall, F1 and AUC values of 82.70%, 85.39%, 83.91% and 0.8424, respectively, without any signs of overfitting.

Three issues will be the focus of future research. Initially, the model architecture and parameters should be adjusted to achieve higher precision and recall values. Furthermore, the dataset should be expanded by collecting more molecules, including a 3D representation, or by using Data Augmentation techniques and Generative Adversarial Networks to to expand the dataset. Lastly, it is important to explore the use of Large Language Models in predicting the properties of chemicals.

## Data Availability

The datasets used and analysed during the current study available from the corresponding author on reasonable request.
